# *Candida spp.* and gingivitis in children with nephrotic syndrome or type 1 diabetes

**DOI:** 10.1186/s12903-015-0042-6

**Published:** 2015-05-08

**Authors:** Dorota Olczak-Kowalczyk, Beata Pyrżak, Maria Dąbkowska, Małgorzata Pańczyk-Tomaszewska, Grażyna Miszkurka, Izabela Rogozińska, Ewa Swoboda-Kopeć, Dariusz Gozdowski, Angelika Kalińska, Anna Piróg, Małgorzata Mizerska-Wasiak, Maria Roszkowska-Blaim

**Affiliations:** Department of Paediatric Dentistry, Medical University of Warsaw, 18 Miodowa St, 00-246 Warszawa, Poland; Department of Paediatric Endocrinology, Medical University of Warsaw, Warszawa, Poland; Department of Dental Medical Microbiology, Medical University of Warsaw, Warszawa, Poland; Department of Paediatric Nephrology, Medical University of Warsaw, Warszawa, Poland; Department of Experimental Statistics and Bioinformatics, Warsaw University of Life Sciences, Warsaw, Poland

**Keywords:** Oral cavity, Immunosuppression, Nephrotic syndrome, Type 1 diabetes, *Candida spp*, Hydrolytic enzyme activity, Gingivitis

## Abstract

**Background:**

Diabetes and Nephrotic syndrome (NS) promote plaque-related gingivitis and yeast-like fungal infections.

The study assesses the impact of *Candida spp*. and general disease- or treatment-related factors on plaque-related gingivitis severity in children and adolescents with Nephrotic syndrome /diabetes.

**Methods:**

Body mass index (BMI), BMI standard deviation score, and oral cavity (Plaque Index – PLI, Gingival Index – GI, mucosa status, presence and Candida enzymatic activity) were assessed in 96 patients (32 with NS: 30- immunosuppressive treatment, 35 - type 1 diabetes, and 29 generally healthy), aged; 3–18 years. Laboratory included cholesterol and triglyceride measurements; in diabetic subjects– glycated haemoglobin, in NS: total protein, albumin, creatinine, haemoglobin, haematocrit, white cell count, urinary protein excretion. Medical records supplied information on disease duration and treatment. A statistical analysis was performed; Kendall Tau coefficient, chi-square test, t-test, and multiple regression analysis ( P < 0.05).

**Results:**

*Candida spp*. often occurred in healthy patients, but oral candidiasis was found only in the NS and diabetes groups (9.37% and 11.43%). Gingivitis occurred more frequently in patients with NS/diabetes. Gingivitis severity was correlated with PLI, age, and yeast enzyme activity in NS – to with immunosuppressive treatment with >1 drug, drug doses, treatment duration, lipid disorders, and BMI; in diabetes, with blood glucose and glycated haemoglobin >8%.

**Conclusion:**

Poor hygiene control is the main cause of gingivitis. Gingivitis severity is most likely related to age, lipid disorders and increase in body mass. *Candida spp.,* in uncompensated diabetes and in those using immunosuppressive treatment, might intensify plaque-related gingivitis.

## Background

Both diabetes and immunosuppressive treatment predispose children to plaque-related gingivitis and oral yeast infections [1-12]. Both diseases present a number of factors promoting bacterial and fungal infections. Reduced salivary flow and modified salivary composition might occur in uncompensated diabetes [13]. Neutrophil activity and cell-mediated immunity are impaired. Patients with diabetes are even considered to present a modified oral bacterial flora, which might promote fungal infections [14,15]. An impaired cellular response is also related to long-term immunosuppressive treatment in Nephrotic syndrome. The syndrome (NS) is a clinical condition with a proteinuria level exceeding the body’s compensating abilities (protein loss over 50 mg/kg/day). Proteinuria results in hypo- and dysproteinaemia, hyperlipidaemia, and modifications in immunoglobulin composition (including decreased IgG levels), which additionally impair the body’s immunity [16].

Candida spp. often colonises the oral cavity without presenting any lesions. However, candidiasis symptoms also occur in an impaired defence mechanism and fungus-host imbalance [17-19]. Yeast-like fungi also frequently found in non-candidiasis related oral mucosal lesions [20,21]. They may cause gingivitis, developing independently out of plaque, such as linear gingival erythema (LGE) manifesting as gingival margin erythema. It may also sometimes develop from the attached related gingiva, and manifest as spontaneous bleeding [4,22].

Yeast-like fungi might also lead to periodontitis. *Candida albicans* has been isolated from periodontal pockets and root canals [23-25]. The production of inflammatory cytokines is said to be more important in the presence of *C. albicans* as a response to cellular wall lipopolysaccharide activity, and thus increasing the severity of periodontitis [26,27]. *Candida albicans* has also been found to facilitate the *P. gingivalis’* invasion into epithelial cells and fibroblasts [28].

Detection of yeast-like fungi does not prove their role in lesion formation. It is crucial to determine their invasive capacity, reflected in the activity of hydrolytic enzymes produced by yeast-like fungi, including alpha–glucosidase (E16) and β-N-acetylglucosaminidase (E18) [29-33]. E16 and E18 were proven to inhibit neutrophil migration to the source of the infection [33]. Beta-N-acetylglucosaminidase has also been found to induce filamentation, i.e. it exerts it impact on the formation of ‘germ tubes’ [32]. An important activity of these enzymes at the lesion site might reflect the pathogenic role of yeast-like fungi.

Children and teenagers with diabetes and with NS undergoing immunosuppressive treatment are predisposed to gingivitis. *Candida spp*. has often colonises the oral cavity, and candidiasis occurs more frequently than in the general healthy population. In the previous study, *Candida spp*. was isolated in 45.4% of children with nephrotic syndrome undergoing immunosuppressive treatment, and in 14.8% of those with type 1 diabetes. Oral candidiasis was diagnosed in 13.3% of patients with NS and in 11.1% of those with uncompensated diabetes [4]. However, even though yeast-like fungi might intensify the infection, their impact on the condition of the gingiva in those diseases has not been assessed.

The present study has assessed the impact of *Candida spp*. and the general factors related to the disease or its treatment in the severe plaque-related gingivitis in children and adolescents with diabetes or Nephrotic syndrome.

## Methods

The study was conducted using 96 subjects: 32 with NS, 35 with type 1 diabetes, and 29 that were generally healthy (control group) (Table 1). The inclusion criteria were: age from between 3 to 18 years and a written informed consent of the child and/or their parent/guardian, as well as:

- In the NS group: no treatment with drugs other than those used in the treatment of NS, no chronic disease other than kidney disease
- In the diabetes group: no chronic disease other than diabetes or drug therapy
- In the control group: no chronic disease or drug therapy

**Table 1:** **Age and sex of patients qualified to the study and biochemical characteristics of children with nephrotic syndrome or type 1 diabetes**

**Group**	**Number of patients (girls/boys)**	**Age in years range (mean ± SD)**	**Biochemical parameter**	**Mean ± SD**
Control group	29 (10/19)	3-17.1 (11.52 ± 4.01 )	-	-
Nephrotic syndrome	32 (13/19)	3-18 (10.05 ± 4.8)	Proteinuria (mg/kg/day)	73.2 ± 104.24
			Albumin (g/dL)	3.54 ± 0.84
			Total protein (g/dL)	6.4 ± 1.17
			Haemoglobin (g%)	13.73 ± 1.46
			Hematocrit (%)	41.74 ± 4.37
			Leukocyte count (x103/mm^3^)	9.8 ± 3.16
			Cholesterol (mg/dL)	262.29 ± 127.33
			Triglycerides (mg/dL)	176.33 ± 165.23
Type 1 diabetes	35 (17/18)	6-17.9 (13.24 ± 2.64)	Blood glucose level (mg/dL)	173.94 ± 70.3
			HbA1c (%)	8.93 ± 2.16

The exclusion criteria included: tobacco smoking, orthodontic appliances or dental prosthesis use, antifungal treatments, and antibiotic therapy at the time of qualification for the study and during the three previous weeks. The NS and diabetes groups included patients referred to the dentists by paediatricians and those who presented themselves for a check up at a nephrology or diabetes clinic. Patients presenting for the first time at the Department of Paediatric Dentistry were qualified to participate in the control group.

The Warsaw Medical University Committee for Ethics approved the study. The study was prospective and included a general medical and oral examination (clinical assessment and laboratory tests) and a medical history review. The oral examination was performed following the general medical examination (clinical and sampling for the scheduled laboratory tests) on the same or following day. Just prior to the clinical the obtained swabs were sent for mycological testing. The dentists were only aware of the patient’s disease (diabetes or Nephrotic syndrome). They did not know the general medical details of the NS and diabetes patients, including the main disease course and treatment plan, neither of the occurrence of other diseases. The statistical analysis of the obtained results was also performed.

### General medical examination

**BMI** was calculated in kg/m^2^ for all clients, and adjusted to their age and gender to provide a BMI standard deviation score (SDS) [34].

#### NS group

Information on the NS patient treatment was obtained from available medical records, including that on the administered drugs with dosage and treatment duration. Laboratory blood tests included total protein (reference range 6–8 g/dL), albumin (reference range 3.7–5.6 g/dL), creatinine, cholesterol, triglycerides, haemoglobin, haematocrit (reference ranges, depending on age), and white blood cell count, measured with standard laboratory methods. A 24-hour urinary protein excretion was measured using turbidimetry.

#### Diabetes group

Information on diabetes duration was obtained from the available medical records. Laboratory data, including measurements of glycated haemoglobin – HbA1c (with D-10 high-performance liquid chromatography), blood glucose levels (with a testing strip using a standard glucose metre), cholesterol and triglycerides, using standard laboratory methods, were collected. The criteria for compensated diabetes were: HbA1c <7% – good, 7–8% – average, and >8%– bad.

### Oral assessment included the oral mucosa, general hygiene level, and gingiva

Oral candidiasis was assessed according to the World Health Organisation guidelines [35]. The oral hygiene score was based on the Silness and Löe plaque index (PLI). Plaque deposits were assessed in the cervical area of six teeth: 16, 12, 24, 36, 32, 44 (55, 51, 63, 75, 71, 83 in deciduous teeth), according to the criteria: 0 – no plaque, 1 – a film of plaque visible on probing the gingival margin, 2 – visible accumulation of deposits, 3 – abundance of soft matter within the gingival pocket [36]. The index is the quotient of the sum of the values obtained for all studied tooth surfaces (medial, distal, buccal, and lingual) and the number of examined areas. The gingival score was based on the Silness and Löe Gingival Index (GI). The gingival tissues were assessed around the same teeth according to the following criteria:

0. No inflammation – healthy gingiva
1. Mild inflammation – slight change in colour and in tissue structure; no bleeding on probing
2. Moderate inflammation – visible glazing, redness, oedema, and hypertrophy; bleeding on pressuring or probing,
3. Severe inflammation – marked redness and hypertrophy; tendency towards spontaneous bleeding; ulceration.

The GI is the quotient of the sum of the values for all gingival surfaces of the scored teeth and the number of scored surfaces [36]. Gingivitis severity was scored as follows: 0.1–1.0: mild inflammation; 1.1–2.0: moderate inflammation, and 2.1–3.0: severe inflammation. Linear gingival erythema, gingival overgrowth and pockets deeper than 4 mm (with the use of a calibrated periodontal probe) were also reported.

### Microbiological examination

Oral swabs from the patients were cultured on Sabouraud agar. After a 24–72 h incubation at 30°C, the following items were assessed:

- Species identification with commercial tests ID 32 C, performed with automated ATB Expression [20],
- Assessment of enzyme activity: valine arylamidase (E7), α-glucosidase (E16), β-N-acetyl-glucosaminidase (E18), performed with API 20 C AUX and API ZYM tests [37,38].

The API-ZYM micro test using 20 micro-vials included 19 substrates to detect 19 hydrolytic enzymes. The first micro-vial served as a negative control. The second contained a substrate for alkaline phosphatase (E2). Micro-vials 3–5 contained substrates for esterase (C4), esterase lipase (C8), and lipase (C14). Micro-vials 6–10 contained substrates for proteases. Finally, micro-vials 13–20 contained substrates for glycosidases. The micro-vials were filled with 65 μL of urine and incubated for 4–4.5 h at 37°C. Following the ncubation, , one drop of reagent (ZYM A or ZYM B) was added to each micro-vial and the intensity of the colour reaction was read after five minutes, using special tables provided with the kit, with scores from 0 to 5, where 0 corresponded to a negative reaction (0 nmol), 1 to 5 nmol, 2 to 10 nmol, 3 to 20 nmol, 4 to 30 nmol, and 5 to a reaction with maximum intensity, i.e. 40 nmol. Three *Candida albicans* isolates, out of eight cultured strains, were used to test for potentially identical enzymatic profiles.

#### Statistical analysis

For all variables, the mean values and standard deviations (SD) or proportions were calculated. The t-test was used to compare means in the examined groups, and the chi-squared test was used to compare fractions. Correlations between the selected variables were assessed using the Kendall rank correlation coefficient (significance level P < 0.05). The statistical analysis was performed using the Statistica 10.0 software. Correlations between the GI and selected properties were calculated jointly for all groups to determine the ratio of ill and healthy subjects in the population. Correlations were also calculated separately for each group of ill children under study, to check whether the correlation was similar to the one in the entire population of healthy and ill subjects. It was necessary to double-check them in order to avoid the Simpson’s paradox, i.e. the effect of apparent correlations resulting from important disparities among the assessed groups. Moreover, a multiple regression analysis was used to assess the simultaneous effect of many independent variables on the GI. Partial standardised regression coefficients were presented.

## Results

### Medical status

The BMI in the NS group ranged between 14.36 and 41.0 kg/m^2^ and the BMI SDS was between −0.9 and 3.3 (mean BMI: 22.1 ± 6.06 kg/m^2^). In the diabetes group, the BMI ranged between 14 and 32 kg/m^2^, and the BMI SDS between −1.3 and 2.5 (mean BMI: 20.07 ± 3.52 kg/m^2^). In the control group, the BMI ranged between 12.2 and 31 kg/m^2^ for the BMI, and between −2.2 and 2.3 for the BMI SDS (mean BMI: 19.51 ± 4.12 kg/m^2^).

In the NS group, disease duration ranged between 0.5 and 15.67 years (mean duration: 5.06 ± 4.95 years). Thirty patients with NS received immunosuppressive treatment, including single drug immunosuppression in 14 patients (with corticosteroids [CS]), immunosuppression with two drugs in 11 patients (in seven patients, CS and cyclosporin A [CsA], and in four patients, CS and azathioprine), and immunosuppression with three drugs in five patients (with CS, CsA and mycophenolate mofetil [MMF]). Corticosteroids, used by 29 patients (mean dose: 33.25 ± 19.9 mg/day, mean treatment duration: 3.99 ± 4.45 years), and cyclosporin A was used in 10 patients (mean dose: 134.54 ± 48.0 mg/day, mean treatment duration 3.88 ± 2.95 years), were the most commonly administered drugs. Proteinuria occurred in 23 patients with NS (71.87%), hypoalbuminemia in 23 patients (71.87%), decreased serum total protein levels in 10 patients (31.25%), decreased haemoglobin levels in four patients (12.5%), increased haematocrit in 16 patients (50.0%), hyperleukocytosis in 15 patients (46.87%), elevated cholesterol levels in 22 patients (68.75%), and elevated triglyceride levels in 14 patients (43.75%).

In patients with diabetes, the duration of the disease ranged between 0.1 and 9.58 years (mean duration: 2.82 ± 2.5 years). Patients with diabetes received from 0.1 to 1.3 units of insulin/kg of body mass (mean 0.76 ± 0.29). Blood sugar level ranged between 69 and 300 mg/dl. In 17 patients (48.57%), the Hb1c level was higher than 8% (uncompensated diabetes) and in four patients (11.4%), it was lower than 7%. Hypercholesterolemia occurred in seven patients (20.0%) and higher blood triglyceride levels in five patients (14.28%). Table 1 presents the mean values of the evaluated biochemical parameters in patients with NS, as well as the levels of glycated haemoglobin (HbA1c).

### Oral cavity

*Candida spp* were frequently found in the oral cavity of the controls (41.37% subjects), and less often in patients with NS (34.37%, all under immunosuppressive treatment) and those with diabetes (22.85%) (statistically insignificant differences). Only *Candida albicans* was isolated. Table 2 presents oral *Candida* prevalence in the respective groups and the activity of the Candida enzymes obtained from the oral cavity, including valine arylamidase (E7), alpha-glucosidase (E16), and N-acetyl-beta-glucosaminidase (E18). A considerably higher level of E16 activity was discovered only on comparing the NS group to the control group (Table 2). Oral candidiasis was diagnosed in three patients with NS (9.37%) and in four with diabetes (11.43%). Erythematous candidiasis, pseudomembranous candidiasis with associated median rhomboid glossitis, and angular cheilitis were resident in patients with NS. Erythematous candidiasis (1), angular cheilitis (3), and pseudomembranous candidiasis with associated median rhomboid glossitis were noted in patients with diabetes.

**Table 2:** **Candida spp. prevalence and activity of Candida enzymes isolated from the oral cavity in patients from the control group (C), with Nephrotic syndrome (NS), and with type 1 diabetes (D1)**

**Group**	**Candida spp.**	**Activity of Candida enzymes isolated from oral cavity**							
		**Code E7 or E16 or E18**		**Code E7**		**Code E16**		**Code E18**	
		**1-5**	**5**	**1-5**	**5**	**1-5**	**5**	**1-5**	**5**
	**n/%**			**n**					
NS	11/34.37	11/34.37	8/25.00	11	1	9	5	10	6
D1	8/22.86	7/20.0	6/17.14	7	0	7	4	9	4
C	12/41.37	10/34.48	2/6.89	11	0	10	0	10	2
P-value - chi-squared test									
NS vs C	0.573	0.993	0.057	0.773	0.337	0.592	0.026*	0.788	0.171
D1 vs C	0.111	0.192	0.217	0.112	1.000	0.192	0.060	0.445	0.536

*significantly different at P < 0.05.

Gingivitis was more frequent in patients with diabetes (80%) and with NS (62.5%) than in the controls (37.93%). There was a statistically significant difference only between D1 and C (Table 3). No patient presented severe gingivitis or periodontitis. Nine NS patients presented periodontal pockets deeper than 4 mm in a single tooth, caused by cyclosporine-associated gingival hyperplasia. Moderate gingivitis was diagnosed significantly more often in the NS group. Patients with both diabetes and NS presented significantly higher GIs than did the controls. Gingivitis was related to plaque presence in all the study groups. ab. 3).

**Table 3:** **Oral hygiene (PLI) and gingivae condition (GI) in patients from groups: with nephrotic syndrome (NS), with diabetes (D1), and control (C)**

**Parametry**		**NS**	**D1**	**C**	**P-value**	
PL I	mean ± SD	0.98 ± 0.61	1.02 ± 0.51	0.53 ± 0.69	NS vs C	0.011*
					D1 vs C	0.002*
GI		0.62 ± 0.69	0.47 ± 0.38	0.24 ± 0.42	NS vs C	0.014*
					D1 vs C	0.026*
GI ≥ 0.1	n/%	20/62.5	28/80.0	11/37.93	NS vs C	0.055
					D1 vs C	0.001*
GI ≥ 1.1		10/31.25	4/11.43	1/3.45	NS vs C	0.005*
					D1 vs C	0.236
Correlation between PL I and GI		0.482*	0.633*	0.670*	-	
		(P < 0.001)	(P < 0.001)	(P < 0.001)		

*significantly different at P < 0.05; the t-test was used to compare means, and the chi-squared test to compare fractions.

The Kendall rank correlation coefficient for NS and controls, as well as for D1 and controls, also indicated a correlation between GI and yeast enzyme activity (E7, E16, and E18). In diabetes, the occurrence of only yeasts in the oral cavity was crucial (Table 4). The individual assessment of the respective groups indicated that intensified gingivitis in the NS patients Nephrotic syndrome was related only to a high E18 activity. Immunosuppressive treatment, especially with more than one drug, disease duration, the BMI and BMI SDS, a higher blood cholesterol level (mg/dL), and age had a crucial impact on gingivitis severity in patients with NS. Additionally, the duration of glucocorticoid treatment (significant Kendall rank correlation coefficient: 0.258), and the dosage and duration of CsA treatment (significant Kendall rank correlation coefficient: 0.461, 0.407) were also correlated with the GI. The assessment in D1 and controls indicated a correlation between GI and age, as well as disease duration and elevated blood glucose levels. The correlation coefficient was statistically and significantly negative for HbA1c <7%, and positive for HbA1c > 8% (Table 4). A similar assessment only in the diabetes group confirmed a negative correlation for HbA1c <7%. The results of the multiple regression analysis only confirmed the correlation between GI and immunosuppressive treatment and blood cholesterol levels in NS patients (partial standardised coefficients of multiple regression: 0.421 and 0.391). The coefficient of determination (R^2^) was between 0.309–0.662 (Table 4).

**Table 4:** **Kendall Tau correlation coefficients (KTC) and partial standardized coefficient of multiple regression (MR) between GI and** ***Candida spp*** **. occurrence, candidal enzymatic activity in oral cavity, and systemic factors (immunosuppressive treatment, selected clinical and biochemical parameters) in the NS and C, NS, D1 and C, and D1 groups**

**Parameters**		**NS and C**		**Only NS**		**D1 and C**		**Only D1**	
		**KTC**	**MR**	**KTC**	**MR**	**KTC**	**MR**	**KTC**	**MR**
*Candida spp.* occurrence		0.046	−0.042	0.082	0.080	0.180*	−0.341	0.243*	−0.708
Enzymatic activity of Candida spp. isolated from the oral cavity	E7	0.222*	0.189	0.098	0.053	0.272*	0.211	0.463	
	code 5	−0.010	−0.343*	−0.060	−0.344	*-*		-	
	E16	0.215*	0.385	0.070	−0.220	0.316*	0.678	0.417*	0.424
	code 5	0.094	−0.403	0.025	0.083	0.283*	−0.173	0.377*	−0.065
	E18	0.259*	−0.143	0.134	−0.377	0.260*	−0.067	0.295*	0.648
	code 5	0.273*	0.045	0.246*	0.583	0.229*	0.085	0.236*	−0.329
Immunosuppressive treatment		0.205*	−0.216	0.136	0.154	-	-	-	-
	>1 drug	0.359*	0.421*	0.388*	0.465	-	-	-	-
Elevated cholesterol level		0.219*	0.391*	0.201	0.473*	0.038	-	−0.137	-
Elevated triglyceride level		0.166	0.030	0.133	−0.067	0.115	-	−0.061	-
HbA1c		-	-	-	-	0.300*	0.145	0.207	0.128
	<7%	-	-	-	-	−0.328*	−0.282	−0.252*	−0.218
	>8%	-	-	-	-	0.255*	−0.045	0.189	−0.016
Blood glucose level (elevated)		-	-	-	-	0.187*	−0.098	0.026	−0.065
BMI		0.329*	0.289	0.302*	−0.062	0.141	0.057	−0.105	0.168
BMI in SDS		0.240*	−0.082	0.268*	0.191	0.030	−0.042	−0.067	−0.281
Disease duration		0.226*	0.136	0.256*	0.197	0.200*	−0.063	−0.041	−0.033
Age		0.195*	0.033	0.151	0.003	0.201*	0.100	−0.052	0.000
Sex		−0.006	0.110	0.149	0.340	−0.038	−0.056	0.131	0.006
R^2^ – coefficient of determination		-	0.468	-	0.662	-	0.309	-	0.520

*statistically significant correlation (P < 0.05).

Since the analysis was performed only for the control group, it indicated a correlation between the GI and the BMI (statistically significant Kendall rank correlation coefficient: 0.378). The analysis performed in all 96 patients also confirmed a positive correlation between the GI and the BMI (statistically significant Kendall rank correlation coefficient: 0.204). However, correlations with the BMI SDS were not statistically significant.

## Discussion

There are many publications associating frequent gingivitis and oral candidiasis with impaired immune defence mechanisms, such as in organ recipients receiving immunosuppressive treatment, cancer patients receiving cytotoxic treatment, patients with AIDS, or patients with diabetes [1-12]. In the present study, candidiasis only occurred in children with NS or diabetes. In those groups, gingivitis was more frequent d and showed a more severe course compared to that in the generally healthy children. Dental plaque was the main cause behind gingivitis. The present study confirmed a correlation between GI, PLI, and the child and adolescent age. Romero et al. also established that GI increased with age in children, even though PLI decreased [39].Treatment of gingivitis consisting in removal of the dental plague and factors contributing to its retention may be not successful in patients with systemic disease. Systemic diseases are usually associated with diverse disorders which may favourably affect the development of gingivitis or increase its severity accompanied by diverse disorders which may favourably affect the presence or/development of gingivitis. Among them, there may be nonmodifiable and modifiable factors whose influence (=activity) may be nonmodifiable and modifiable factors, whose influence (activity) may be an may decrease their effect and consequently achieve better more effective results.

The present study, assessing the impact of the respective factors, including the occurrence if *Candida spp.* in the oral cavity, and the systemic disorders in diabetes and NS, selects factors associated with the disease or it’s treatment influencing severity of gingivitis that may intensify development. The study draws attention to considering antifungal treatment as element of gingivitis therapy. It accentuates the significance of maintaining a proper body weight. The assessment of the simultaneous impact of factors (multiple regression) was not statistically significant for most of them. It was caused of their mutual correlations. However, the coefficients of determination (R^2^) indicated a significant impact of the assessed factors on GI (Table 4).

Gingiva in children with NS has not been previously assessed, however, the observation of patients with kidney or liver transplants that are under immunosuppressive treatments indicated a correlation between gingivitis severity and GI [20,21]. Other authors concluded the same in diabetes, independent of the definition of periodontal disease [12,40-42]. Lalla et al., examining 83 patients with diabetes and 80 generally healthy children and adolescents, confirmed that children with diabetes presented significantly more dental plaque and a higher GI than did the nondiabetic controls (plaque index 1.2 vs. 1.1, gingival index 1.2 vs. 1.1, respectively) [12]. Lalla et al. used three definitions of periodontal disease: attachment loss, gingival bleeding, or both. Their regression analysis indicated that diabetes presented a statistically significant correlation with periodontitis, even in the 6- to 11-year-old group of patients with diabetes. However, they did not find any correlation between the inflammation of periodontal tissues and the degree of diabetes compensation (mean HbA1C) and lipid profiles [12]. This is contrary to the present study and to earlier evidence suggesting improper management of diabetes and the associated metabolic disorders predisposed to more severe periodontitis [43]. Saes Busato et al. confirmed, in their study on young patients with diabetes, with the results based on the Community Periodontal Index (CPI), the negative impact of improper diabetes management. Adolescents with poor metabolic control presented a higher CPI than did the group with good metabolic control (2.0 and 1.4, respectively) [42]. Lalla et al. noticed a correlation between periodontitis in diabetes and BMI, which was also confirmed by others authors that used adults in their studies [12,44,45]. The results of the present study also suggest a correlation with gingivitis severity. However, it was only evident for all of the patients together, independently of their general health, and in controls and NS.

The correlation between GI and high cholesterol levels in children with nephrotic syndrome is worth noting. A higher level of cholesterol and triglycerides occurs in nephrotic syndrome relapse and can be upheld for a long period of time, even in remission [46]. Hyperlipidaemia in remission, and especially total cholesterol level in the serum, is considered a predictor of idiopathic nephrotic syndrome relapse in children [47]. Patients with diabetes also present higher cholesterol and triglyceride (TRG) levels, even if the blood glucose level was well-managed. Periodontitis-induced bacteraemia is also implied increased serum pro-inflammatory cytokine levels, leading to hyperlipidemia [11]. Although the present study did not confirm correlations between the GI and lipid profile disorders in diabetes, it is necessary to continue studying both diseases in larger patient groups.

There many factors promoting periodontitis in diabetes (such as alterations in host response, collagen metabolism, vascularity, gingival crevicular fluid, and heredity patterns) and in those receiving immunosuppressive treatment (drugs, dosage, and treatment duration). However, the correlation between gingivitis severity and yeast-like fungi in the oral cavity was not assessed, even if fungal infections occurred more often in these patients. In the present study, yeast-like fungi less frequently colonised the oral cavity of patients with nephrotic syndrome or diabetes, compared to the generally healthy participants, but *Candida spp.* more often led to candidiasis. There was no difference between the enzymatic activity of the strains isolated from the oral cavity in patients with NS and diabetes and that of the controls. Plomer-Niezgoda et al. showed different *Candida* enzymatic biotypes, according to the Williamson classification, in patients under immunosuppressive therapy (biotypes C and B) and healthy subjects (biotypes A and B), but both groups presented E7, E16, and E18 activity [48]. It is also interesting to note that the present study established a correlation between gingivitis and the local Candida enzyme oral activity, including valine arylamidase (E7), alpha-glucosidase (E16), and N-acetyl-beta-glucosaminidase (E18). In Kurnatowska and Kurnatowski’s study, 40% of *Candida albicans* strains isolated in gingivitis were biotype F, according to the Williamson classification, i.e. characterised by esterase (E3), E7, naphthol-AS-BI-phosphohydrolase (E12), and E16 activity, 15% were biotype A (also presenting E18 activity), 5% biotype B (E3, E12, E16, and E18 activity), 5% biotype E (E3, E7, and E12 activity), and 5% were biotype G (E3, E12, and E16 activity). The remaining strains were classified as biotypes defined by those authors, including 15% as biotype N (E3 and E18 activity), 5% as biotype I (E18 activity only), and 5% as biotype K (E3, E7, and E16 activity) [49]. These results do not only confirm a correlation between gingivitis and E7, E16, and E18 activity, but also indicate that the N-acetyl-beta-glucosaminidase activity is related to inflammation severity. Considering the role of E18 in the formation of invasive germ tubes, these results may confirm the etiopathogenetic role of *Candida* in the development of gingivitis.

## Conclusions

The main cause of gingivitis is poor oral hygiene. There is most likely a correlation between gingivitis severity and age, BMI and lipid disorders. *Candida spp*., residing in the oral cavity of patients with uncompensated diabetes or those receiving multi-drug immunosuppressive treatment, may potentiate the severity of plaque-related gingivitis.

## Abbreviations

BMI: Body Mass Index
BM/SDS: BMI standard deviation score
PLI: Silness and Löe plaque index
GI: Silness and Löe Gingival Index
HbA1c: Glycated haemoglobin
NS: Nephrotic Syndrome
LGE: Linear Gingival Erythrema
E16: Alpha-glucosidase
E18: β-N-acetylglucosaminidase
E7: valine arylamidase
E2: Alkaline phosphatase
C4: Micro-vials 3–5 contained substrates for esterase
C8: Esterase lipase
C14: Lipase
SD: Standard deviations
CS: Corticosteroids
CsA: Cyclosporine
MMF: Mycophenolate mofetil
AIDS: Acquired Immune Deficiency Syndrome
CPI: Community Periodontal Index
TRG: Cholesterol and Triglyceride
E3: Esterase
E12: Naphthol-AS-BI-phosphohydrolase
